# Relative Bradycardia in Patients with Mild-to-Moderate Coronavirus Disease, Japan

**DOI:** 10.3201/eid2701.203312

**Published:** 2021-01

**Authors:** Gabriel Yan, Alicia Ang, Sai Meng Tham, Alvin Ng, Ka Lip Chew

**Affiliations:** National University Health System, Singapore

**Keywords:** bradycardia, COVID-19, influenza, coronavirus disease, SARS-CoV-2, severe acute respiratory syndrome coronavirus 2, viruses, respiratory infections, zoonoses, Singapore, Japan

**To the Editors:** Ikeuchi et al. (*1*) described the phenomenon of relative bradycardia in patients as an adjunct to the clinical diagnosis of mild-to-moderate coronavirus disease (COVID-19). Relative bradycardia is defined as an increase in pulse rate of <18 bpm for each 1°C rise in body temperature or a body temperature >38.9°C and pulse rate <120 bpm (*2*). We performed a retrospective study comparing COVID-19 and influenza patients in a tertiary hospital in Singapore. Our study was reviewed and approved by the National Healthcare Group Domain Specific Review Board (reference no. 2020/00324)

We reviewed medical records of patients with COVID-19 or influenza, confirmed by reverse transcription PCR, who were treated during October 2019–April 2020. Patients on β-blockers were excluded (14 COVID-19 patients and 25 influenza patients). Eighty-six patients with COVID-19 and 74 patients with influenza were included; 73 influenza cases were influenza A and 1 influenza B. For COVID-19 patients, median age was 40.6 (range 18–72) years and 49/86 (57%) were male; for influenza patients, median age was 54 (range 22–85) years and 34/74 (45.9%) were male. Fourteen (16.3%) COVID-19 patients and 29 (39.2%) influenza patients had fever >38.9°C; only 4 (13.8%) influenza patients and 0 COVID-19 patients had pulse rates >120 bpm. Median pulse rate was 98.5 (interquartile range 94–101) bpm for COVID-19 patients and 99 (interquartile range 97–116) bpm for influenza patients. Linear regression of the peak temperature and the associated pulse rate of the patient predicted an increase in pulse rate of 11.12 (95% CI 7.65–14.60) bpm for COVID-19 patients and 9.5 (95% CI 5.86–13.14) bpm for influenza patients for each 1°C increase in body temperature.

Our data support the observations by Ikeuchi et al. (*1*) of relative bradycardia in COVID-19 patients. However, results from our cohort demonstrate relative bradycardia in patients with both viral illnesses, indicating that this phenomenon cannot be used to reliably distinguish COVID-19 from influenza and has limited clinical utility in patients who have acute respiratory illnesses.
